# Supplementary material for: Genome-wide CRISPR screen identifies protein pathways modulating tau protein levels in neurons
Source: Commun Biol. 2021 Jun 14;4:736. doi: 10.1038/s42003-021-02272-1 (PMC8203616; doi:10.1038/s42003-021-02272-1)
Supplement: Supplementary file 2 — Supplementary Information [file 42003_2021_2272_MOESM2_ESM.pdf]

Supplementary Information

Genome wide CRISPR screen identifies protein pathways modulating tau protein levels in neurons

Carlos G. Sanchez[1], Christopher M. Acker[1], Audrey Gray[1], Malini Varadarajan[2], Cheng Song[2], Nadire R. Cochran[2], Steven Paula[2], Alicia Lindeman[2], Shaojian An[2], Gregory McAllister[2], John Alford[2], John Reece-Hoyes[2], Carsten Russ[2], Lucas Craig[1], Ketthsy Capre[1], Christian Doherty[1], Gregory R. Hoffman[2], Sarah J. Luchansky[2,3], Manuela Polydoro[1], Ricardo Dolmetsch[1], Fiona Elwood[1*]

[1] Neuroscience, Novartis Institutes for BioMedical Research, 22 Windsor Street, Cambridge, MA 02139, USA

[2] Chemical Biology and Therapeutics, Novartis Institutes for BioMedical Research, 181 Massachusetts Ave, Cambridge, MA 02139, USA

[3] Present address: Vertex Pharmaceuticals, 50 Northern Ave., Boston, MA 02210, USA

*Correspondence to Fiona Elwood at fiona.elwood@novartis.com

Supplementary Figure 1

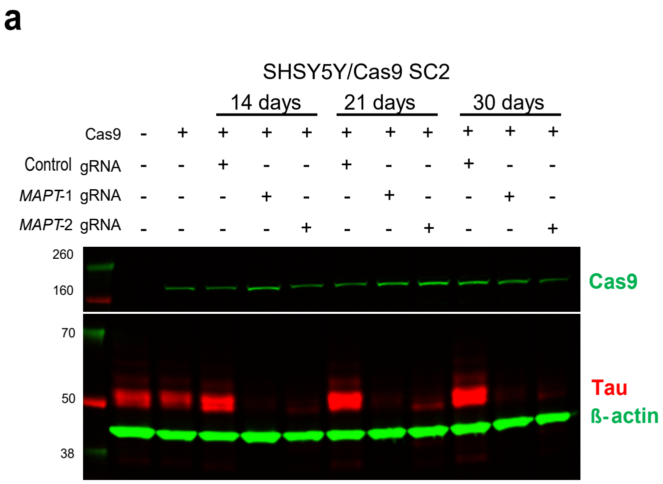

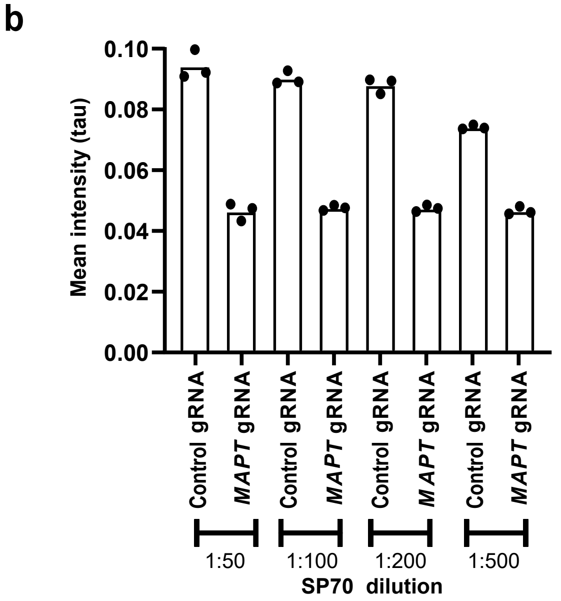

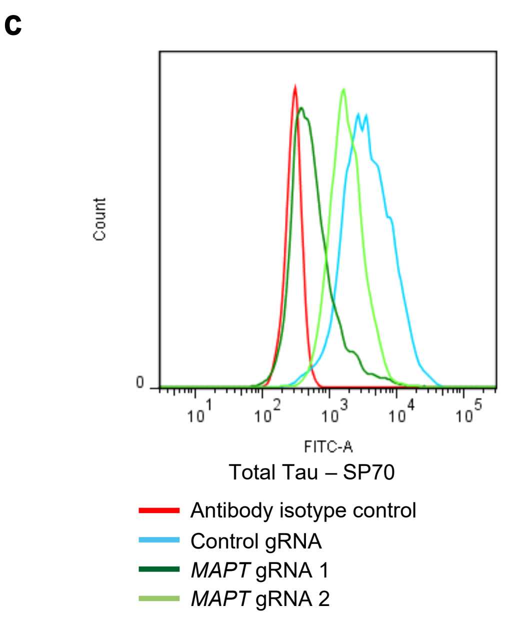

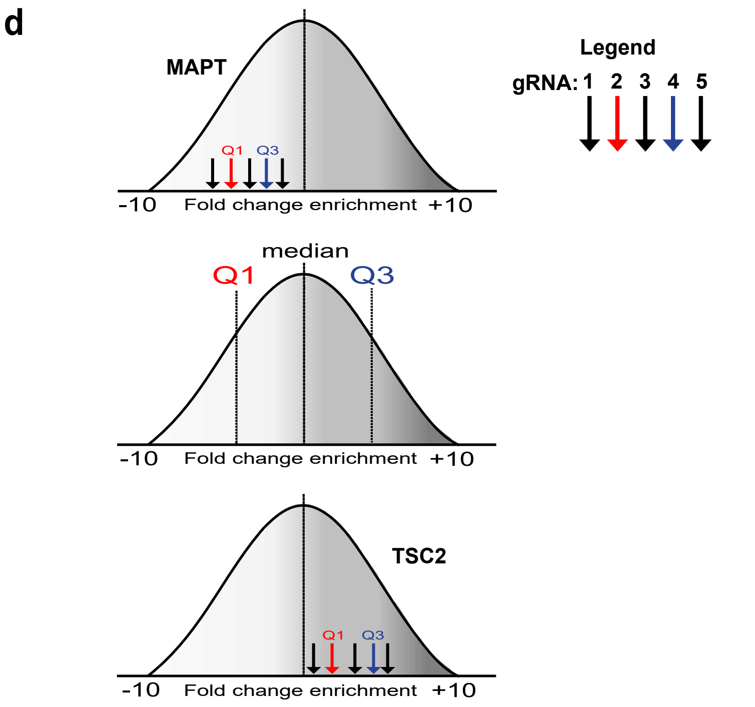

Comparison name	Symbol	Q1	Q3	RSA Dn	RSA Up
SH-SY5Y 30 day edit	MAPT	-5.36	-3.08	-7.48	-0.14
SH-SY5Y 30 day edit	TSC2	2.50	5.05	-0.13	-8.75

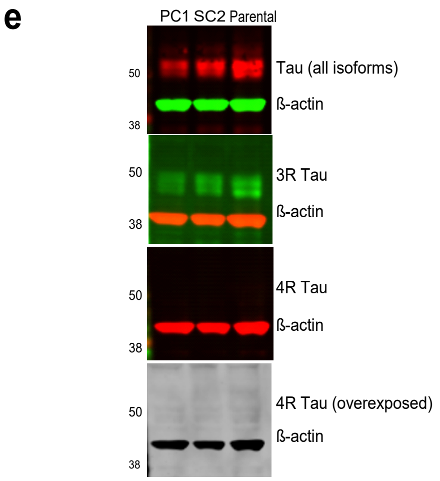

Supplementary Figure 1. Characterization of SH-SHY5Y Clone 2 (SC2) cell lines expressing Cas9.

a) Tau (SP70) and Cas9 western blot analysis on the SH-SH5Y/Cas9 (SC2) cell line, infected with control or *MAPT* gRNAs for 14, 21, or 30 days of editing.

b) Immunofluorescence quantification of different Tau SP70 dilutions on the SH-SH5Y/Cas9 (PC1) cell line infected with lentivirus containing control or *MAPT* gRNA. Data is represented as mean fluorescence intensity ± SEM.

c) Plot of FITC-Area (FITC-A) of cells infected with lentivirus containing control non-target gRNA (light blue) or *MAPT* gRNA (green and light green). Cells were stained with Tau SP70 or an antibody isotype control (red).

d) Fold change enrichment for each of the gRNAs used to knockout *MAPT* and TSC2. Q1 (red) and Q3 (blue) results are noted along a distribution plot. Arrows signify individual gRNAs for each gene. RSA-Redundant siRNA Activity score, Q-Quartile, Dn-Down

e) Total Tau, Tau (3R) and Tau (4R) western blot analysis on the SH-SH5Y/Cas9, PC1 and SC2 cell lines.

Supplementary Figure 2

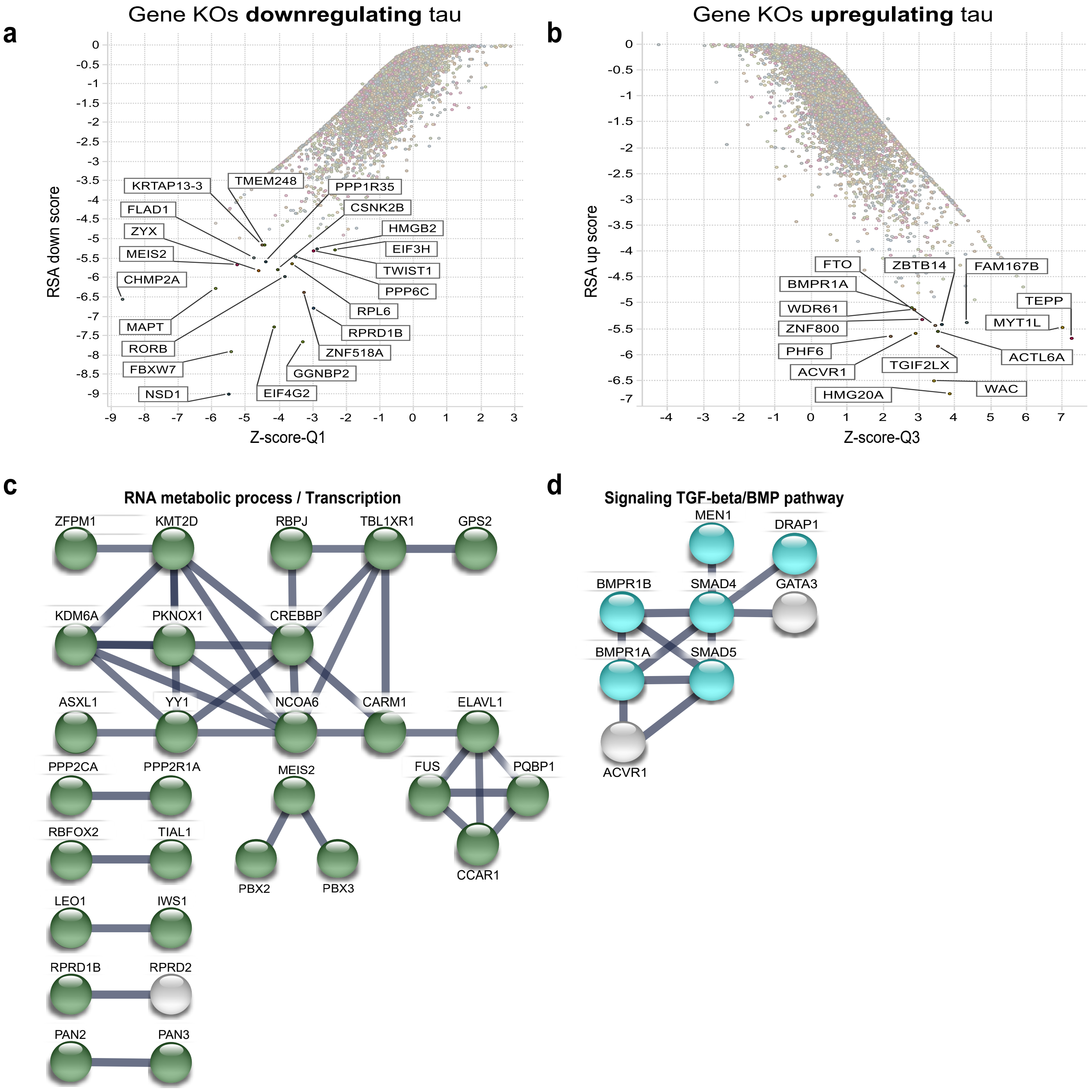

Supplementary Figure 2. Gene knockouts up-regulating and down-regulating tau protein levels in SH-SY5Y cells in genome-wide CRISPR screen.

a, b) Scatter plots, with RSA scores (Y-axis) vs Z-score quartiles (X-axis), for 21 day edited group from primary genome wide screen. Plots shows genes knockouts downregulating (a) and upregulating (b) tau protein levels. Labeled genes are shown for candidate hits with high significance. KO-Knockout, Q-Quartile, RSA-Redundant siRNA Activity score

c, d) STRING enrichment pathways for highly validated genes (primary and mini array screens). Links between nodes are highly confident connections.

c) Additional pathways identified for gene knockouts downregulating tau protein levels.

d) Additional pathways identified for genes knockouts upregulating tau protein levels.

Supplementary Figure 3

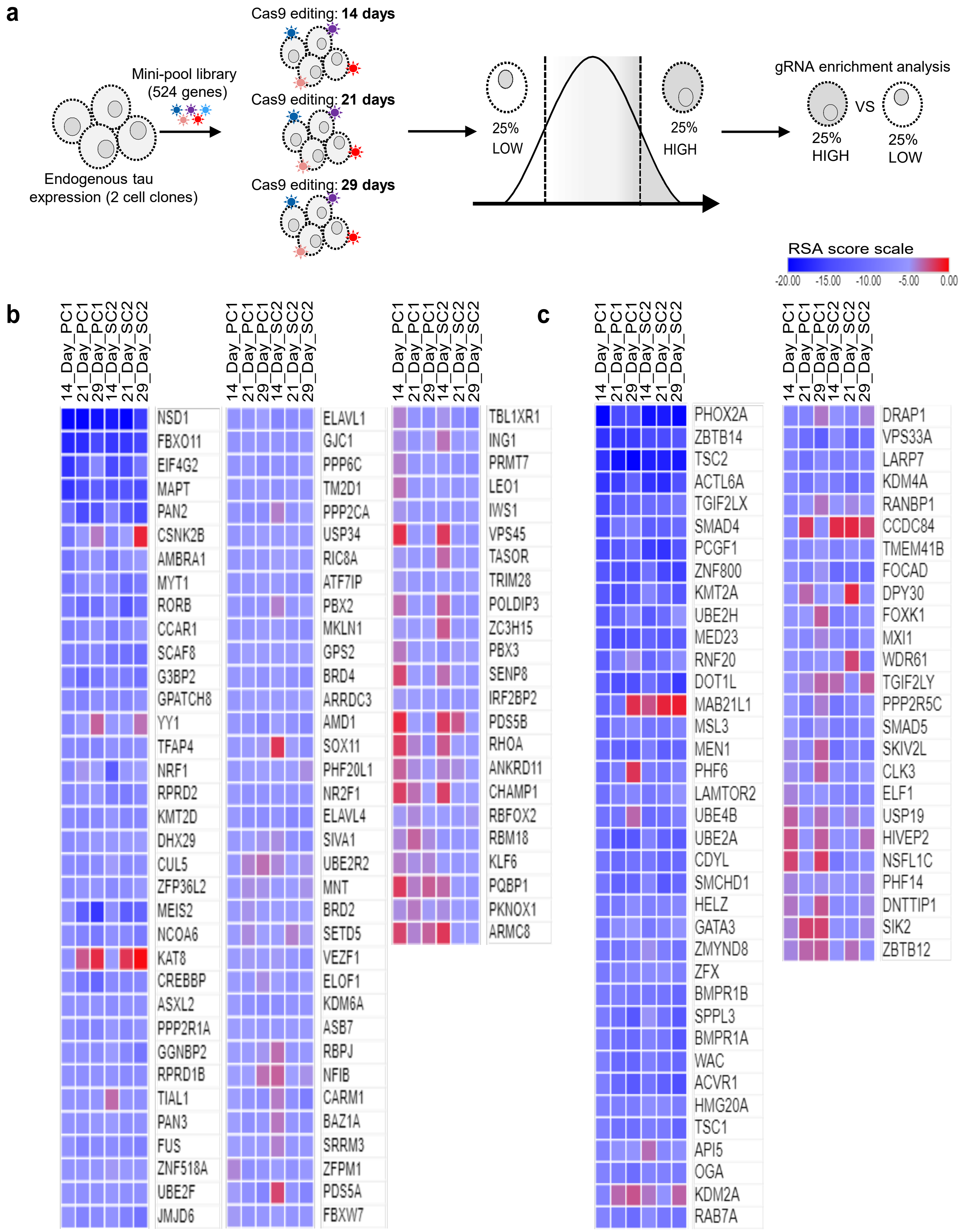

Supplementary Figure 3. Validated Gene knockouts up-regulating and down-regulating tau protein levels in SH-SY5Y cells in mini pool CRISPR screen

a) Schematic representation of validation custom array mini pool screen.

b, c) Heat maps representing validated candidate genes, for gene knockouts downregulating (b) or upregulating tau protein levels (c), with an RSA score of ≤-5.0. RSA-Redundant siRNA Activity score

Supplementary Figure 4

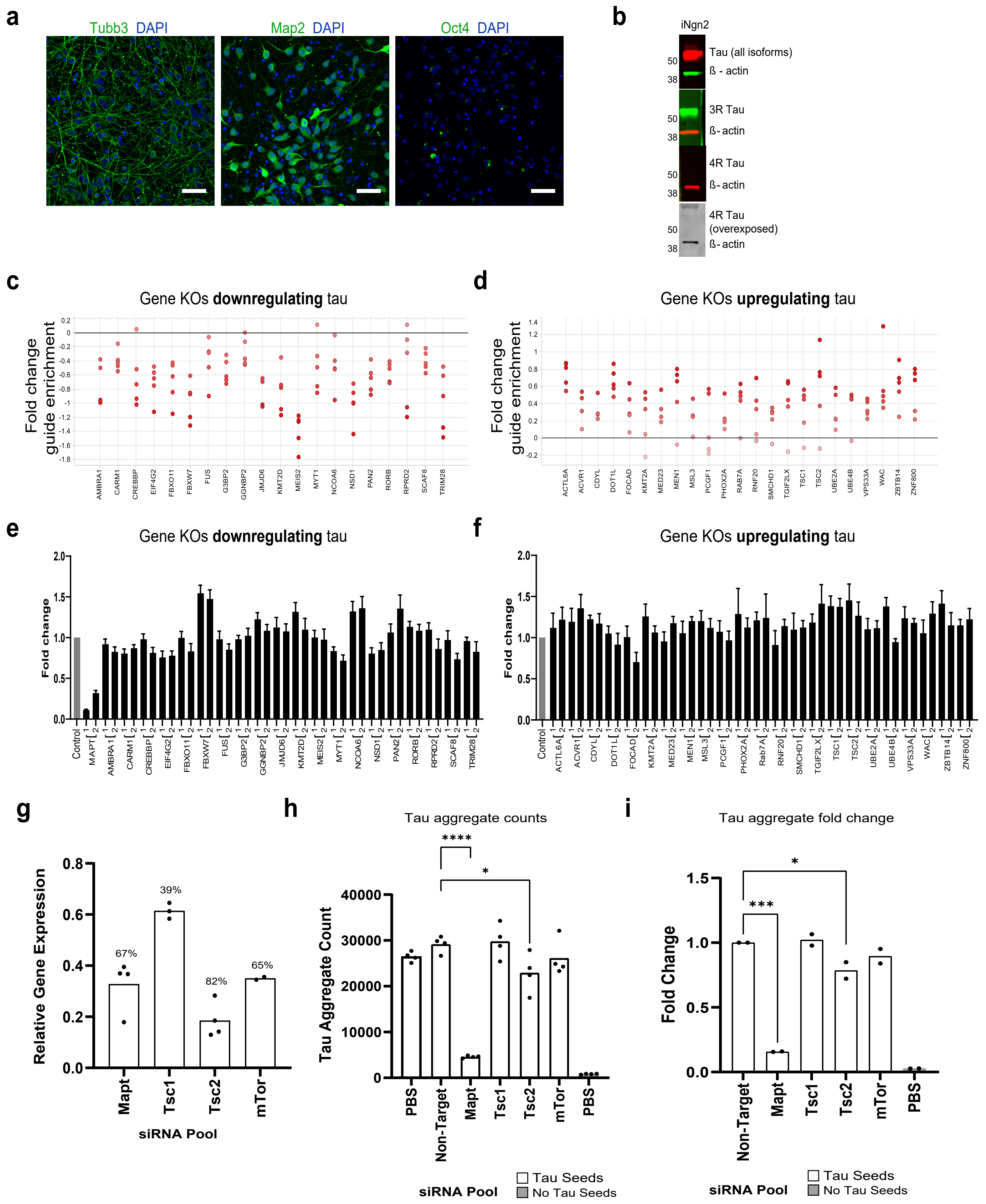

Supplementary Figure 4. Validation of a subset of candidate genes in a human neuronal system

a) Immunocytochemistry of neuronal markers TUBB1 and MAP2; and stem cell marker OCT4 in iNgn2 neuronal cultures 14 days post differentiation. Scale bar represents 1000μm.

b) Total Tau, Tau (3R) and Tau (4R) western blot analysis on the iNgn2 neuronal cultures 14 days post differentiation.

c, d) Performance of 5 gRNAs targeting individual genes from the 30 day edited group from primary genome wide screen. (c) Gene knockouts downregulating and (d) upregulating tau protein levels. Strength of phenotype is noted by stronger shade of red.

e, f) Normalized fold change results of tau protein levels in neurons that were edited using individual gRNAs. Data is represented as mean ± SEM for 7 biological replicates.

g) Relative gene expression of target gene knockdowns for MAPT, Tsc1, Tsc2 and mTor in rat neurons. Respective percent knockdown noted over each bar. Data is represented as mean ± SEM for at least 2 biological replicates.

h) Total tau aggregate object counts in neuronal knockdowns of just PBS or non-target, MAPT, Tsc1, Tsc2 and mTor. White and grey bars show neurons incubated with tau seeds and no tau seeds, respectively. Data is represented as mean ± SEM for 4 biological replicates. One-way ANOVA was performed; **** is P-value of <0.0001 as compared to non-target control.

i) Fold change of tau aggregates normalized to non-target controls in neuronal knockdowns of just PBS or non-target, MAPT, Tsc1, Tsc2 and mTor. White and grey bars show neurons incubated with tau seeds and no tau seeds, respectively. Data is represented as mean ± SEM for 2 biological replicates. One-way ANOVA was performed; *** is P-value of 0.0001 as compared to non-target control.

Supplementary Figure 5

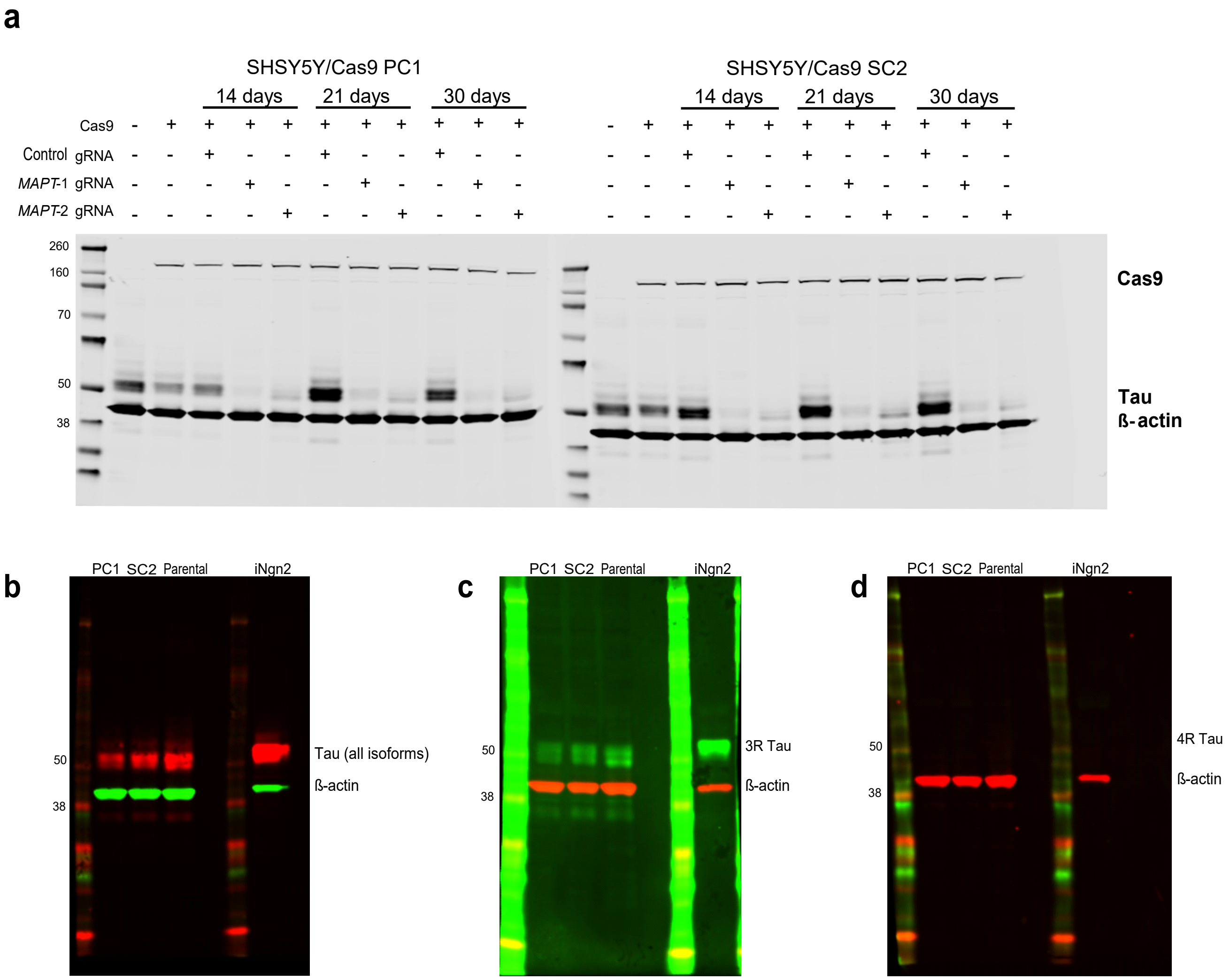

Supplementary Figure 5. Characterization of SH-SHY5Y Pool (PC1), Clone 2 (SC2) and iNgn2 cell lines expressing Cas9. Unedited western blot analysis.

a) Tau (SP70) and Cas9 western blot analysis on the SH-SH5Y/Cas9 PC1 and SC2 cell lines, infected with control or *MAPT* gRNAs for 14, 21, or 30 days of editing. Uncropped Figure 1c and Supplementary Figure 1a.

b) Total Tau, western blot analysis on the SH-SH5Y/Cas9 PC1, SC2 and iNgn2 neuronal cell lines. Uncropped Supplementary Figure 1c and Supplementary Figure 4b.

c) Tau (3R), western blot analysis on the SH-SH5Y/Cas9 PC1, SC2 and iNgn2 neuronal cell lines. Uncropped Supplementary Figure 1c and Supplementary Figure 4b.

d) Tau (4R), western blot analysis on the SH-SH5Y/Cas9 PC1, SC2 and iNgn2 neuronal cell lines. Uncropped Supplementary Figure 1c and Supplementary Figure 4b.

Supplementary Figure 6

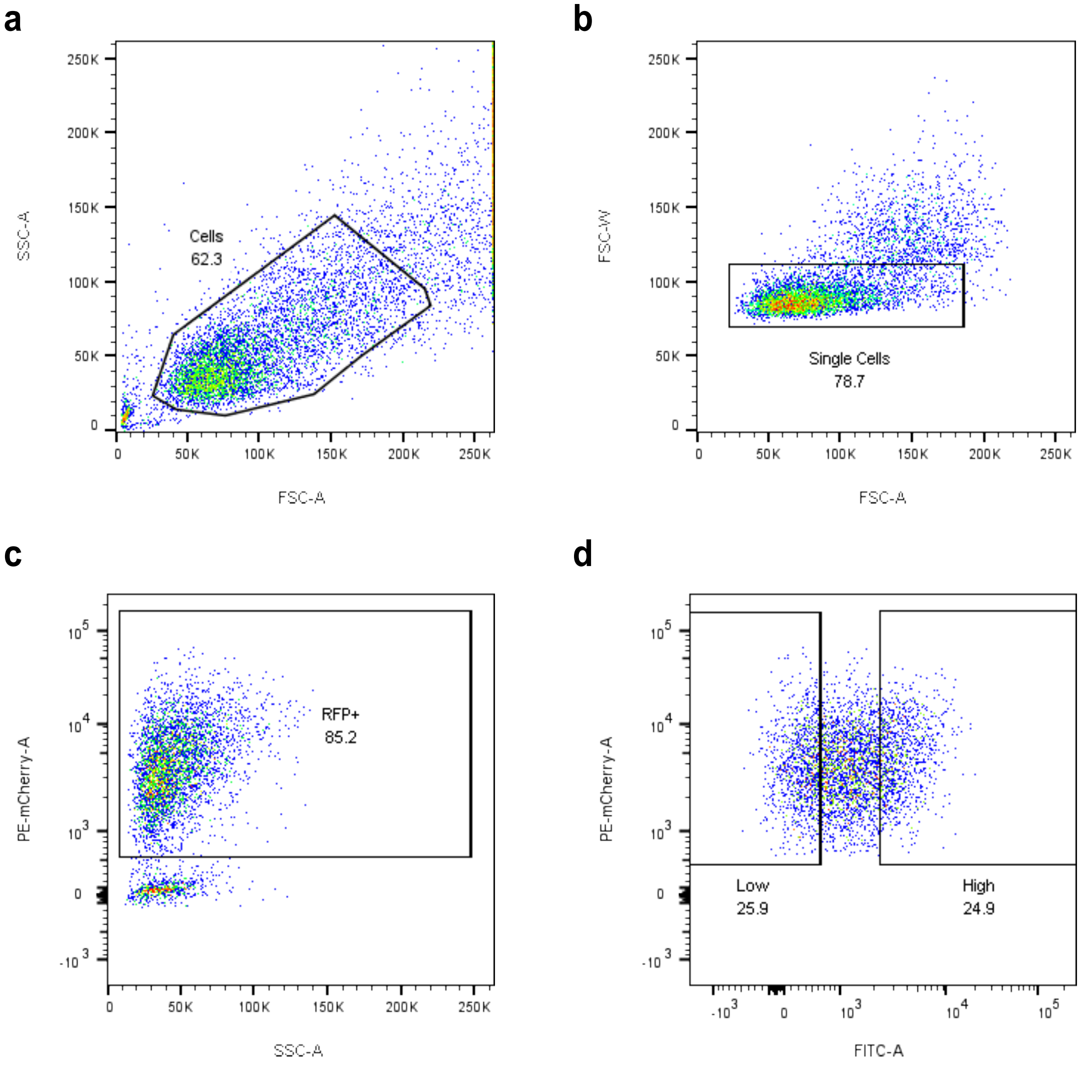

Supplementary Figure 6. Whole genome and mini-pool screening FACS gating strategy for tau protein levels (strategy used for all screening samples)

a) Cells were selected using side scatter area (SSC-A) and forward scatter area (FSC-A).

b) Single cells were then gated from gates in (a) by using forward scatter width (FSC-W) and (FSC-A).

c) gRNA infected cells (RFP positive) were then gated from gates in (b) by using PE-mCherry area (PE-mCherry-A).

d) RFP positive cells were then gated for tau protein levels (FITC-A). 25% of cells were sorted from a population of cells with low FITC-A, and 25% of cells were sorted from a population of cells with high FITC-A.

Supplementary Methods

In vitro rat inclusion assay

Rat cortices were dissociated using a Papain Dissociation System (Worthington Biochemical Corporation, cat#LK003150) following manufacturer's instructions. Briefly cortices were incubated with 1mL of papain solution and 100µL of DNase solution for 10 minutes at 37°C. Tissue settled and solutions were removed and set aside, then cortices were re-incubated using 1.5mL of papain solution and 150µL of DNase solution for 10 minutes at 37°C. Solutions from both time points were combined and tissue was then centrifuged at 1200rpm for 4 minutes. Supernatant was removed and cells were re-suspended in 8mL of Ovomucoid inhibitor and centrifuged at 1200rpm for 4 minutes. Supernatant was removed and cells were re-suspended in Neurobasal Plus Media (ThermoFisher Scientific #A3582901), supplemented with 1% Glutamax (ThermoFisher Scientific #12491), 1% Pen/Strep (ThermoFisher Scientific #15240-062) and 2% B27 Plus (ThermoFisher Scientific # A3582801). Cells were counted and plated in a PDL coated 96-well plate (Corning #354640) at 40,000 cells in 150µL of media per well and incubated at 37°C. 5 days after plating, half the media was changed per well and 1.5uM of Accell siRNA was introduced per well. Accell siRNAs used: Non-Target (Horizon Discovery #D-001910-10-05), *MAPT* (Horizon Discovery #E-089500-00-0005), Tsc1 (Horizon Discovery #E-096662-00-0005), Tsc2 (Horizon Discovery #E-091541-00-0005) and mTor (Horizon Discovery #E-090701-00-0005). 2 days after siRNA addition, 1.5µL of human AD tau seeds was introduced to each of the wells that were incubated with siRNA. 7 Days post seed incubation a half media change was performed. Methanol fixation of tissue cells was then performed 7 days after second half media change (21 days post cell plating). ICC using tau T49 antibody was done and cells were imaged using Incell 6500.

Purification of tau seed from AD brains

AD tau seeds were extracted from human brains by following the purification of tau seed protocol [2]. Briefly, a high salt lysis buffer was prepared using 10mM Tris-HCL pH7.4, 0.8M NaCl, 1mM EDTA, 2mM Dithiothreitol (DTT), 10% Sucrose, 0.1% Sarkosyl, Roche Complete tablets (Cat# 04693159001)

and Roche PhosSTOP tablets (Cat# 04906837001). Buffer was prepared based on tissue weight multiplied by 9µL of lysis buffer. Tissue was homogenized in lysis buffer using a Precellys homogenizer. Tissue was centrifuged at 10,000g for 10 minutes at 4°C. Supernatants were collected and pellet resuspended in 2/3 the volume lysis buffer before re-centrifuging at 10,000g for 10 minutes at 4°C. Supernatants were pooled together and 1% sarkozyl added before incubating for 1 hour at 4°C. The samples were then centrifuged at 300,000g (50,000rpm) for 1 hour at 4°C using a VCM rotor, (Beckman 50.2Ti). The pellet was washed once with PBS and resuspended in PBS. This was sonicated with 20-60 short pulses (0.5s/pulse), and finally centrifuged at 10,000g for 30 minutes at 4°C. The supernatant was the final AD tau seed (sarkozyl insoluble fraction) used in the assay.

Taqman qPCR for knockdown expression

Lysates and multiplex RT-PCR were collected and performed using the FastLane Cell Multiplex Kits (Qiagen #216513) and following manufactures instructions. The following Taqman probes used were: *MAPT* (ThermoFisher Scientific #Rn00691532), Tsc1 (ThermoFisher Scientific #Rn00573107), Tsc2 (ThermoFisher Scientific #Rn00562086), mTor (ThermoFisher Scientific #Rn00693900) and GAPDH (ThermoFisher Scientific #Rn01775763). Percent knockdown for each gene was calculated by determining quantification cycle (C_q) for both the target gene and GAPDH in cells treated with either siRNA targeting gene of interest (*MAPT*, Tsc1, Tsc2, or mTor) or for non-targeting control. Delta C_q (ΔC_q) was determined as the difference between C_q of the target gene minus the C_q of GAPDH. ΔC_q was then exponentially transformed using $2^{-\Delta Cq}$ to determine ΔC_q expression for each targeted gene in siRNA treated samples and non-target controls. Technical replicates were then averaged per gene and normalized to ΔC_q expression of the target gene in a non-target control treated cells ($\Delta\Delta C_q$). Percent knockdown was then calculated for each targeted gene using ($1-\Delta\Delta C_q$) x 100.

Statistics and Reproducibility

Images were analyzed for inclusion counts using Cell Profiler [1]. Inclusions were identified using the Tau T49 intensity and size and nuclear counts were identified using Hoeschst stain. 9 fields were taken per well. Each field was then analyzed to determine inclusion (tau aggregate) count and nuclear counts. All fields per well were then pooled to determine the total number of inclusions and nucleus quantified per well. Background of Tau T49 stain was filtered out by comparing wells with no seed controls and determining how many inclusions are picked up by cell profiler in these wells. The total number of inclusions per well were then normalized to the number of inclusions in non-target control wells to determine fold change relative to non-target controls. This experiment was done in 4 biological replicates and One-way ANOVA test was performed to determine significance.

Supplementary References

1. McQuin C*, et al.* CellProfiler 3.0: Next-generation image processing for biology. *PLoS biology* **16**, e2005970 (2018).

2. Guo JL*, et al.* Unique pathological tau conformers from Alzheimer's brains transmit tau pathology in nontransgenic mice. *J Exp Med* **213**, 2635-2654 (2016).
