# Supplementary material for: Genome-wide CRISPR screen identifies protein pathways modulating tau protein levels in neurons
Source: Commun Biol. 2021 Jun 14;4:736. doi: 10.1038/s42003-021-02272-1 (PMC8203616; doi:10.1038/s42003-021-02272-1)
Supplement: Supplementary file 3 — Description of Additional Supplementary Files [file 42003_2021_2272_MOESM3_ESM.pdf]

Description of Additional Supplementary Files

File name: Supplementary Data 1

Description: Unsorted Raw Analysis

File name: Supplementary Data 2

Description: Whole Genome Screen Results

File name: Supplementary Data 3

Description: Mini-Pool Validation Screen Results

File name: Supplementary Data 4

Description: STRING Enrichment Down Regulators

File name: Supplementary Data 5

Description: STRING Enrichment Up Regulators

File name: Supplementary Data 6

Description: iNgn2 screened genes

File name: Supplementary Data 7

Description: Whole Genome NGS Raw Count Data

File name: Supplementary Data 8

Description: Mini-Pool NGS Raw Count Data

File name: Supplementary Data 9

Description: Source data for plots shown in main Figures.
