# Supplementary material for: Genome-wide CRISPR screen identifies protein pathways modulating tau protein levels in neurons
Source: Commun Biol. 2021 Jun 14;4:736. doi: 10.1038/s42003-021-02272-1 (PMC8203616; doi:10.1038/s42003-021-02272-1)
Supplement: Supplementary file 13 — Reporting Summary [file 42003_2021_2272_MOESM13_ESM.pdf]

nature research

Corresponding author(s): Fiona Elwood

Last updated by author(s): Apr 23, 2021

Reporting Summary

Nature Research wishes to improve the reproducibility of the work that we publish. This form provides structure for consistency and transparency in reporting. For further information on Nature Research policies, see our Editorial Policies and the Editorial Policy Checklist.

Statistics

For all statistical analyses, confirm that the following items are present in the figure legend, table legend, main text, or Methods section.

n/a	Confirmed	
☐	☒	The exact sample size (n) for each experimental group/condition, given as a discrete number and unit of measurement
☐	☒	A statement on whether measurements were taken from distinct samples or whether the same sample was measured repeatedly
☐	☒	The statistical test(s) used AND whether they are one- or two-sided<br>*Only common tests should be described solely by name; describe more complex techniques in the Methods section.*
☐	☒	A description of all covariates tested
☐	☒	A description of any assumptions or corrections, such as tests of normality and adjustment for multiple comparisons
☐	☒	A full description of the statistical parameters including central tendency (e.g. means) or other basic estimates (e.g. regression coefficient) AND variation (e.g. standard deviation) or associated estimates of uncertainty (e.g. confidence intervals)
☐	☒	For null hypothesis testing, the test statistic (e.g. F, t, r) with confidence intervals, effect sizes, degrees of freedom and P value noted<br>*Give P values as exact values whenever suitable.*
☒	☐	For Bayesian analysis, information on the choice of priors and Markov chain Monte Carlo settings
☒	☐	For hierarchical and complex designs, identification of the appropriate level for tests and full reporting of outcomes
☒	☐	Estimates of effect sizes (e.g. Cohen's d, Pearson's r), indicating how they were calculated

Our web collection on statistics for biologists contains articles on many of the points above.

Software and code

Policy information about availability of computer code

Data collection	Graphs were generated in Spotfire TIBCO 10.3.3, PRISM (version 8.0c) and Excel 2016 (16.0.5032.1000)
Data analysis	STRING version 11.0 (https://string-db.org/cgi/input.pl?sessionId=X6pZ8A6uGOU3&input_page_show_search=off)<br><br>Open source code was used for pooled CRISPR screening NGS (Bowtie v1.0.1- Langmead et al., 2009, DESeq2 v1.10.1 - Love et al., 2014).<br><br>Open source code was used for RNA-seq (STAR aligner v2.5.1b, HTSeq-count v0.6.0, RSEM v1.2.28, DESeq2 V 1.16.1 - Love et al., 2014).

For manuscripts utilizing custom algorithms or software that are central to the research but not yet described in published literature, software must be made available to editors and reviewers. We strongly encourage code deposition in a community repository (e.g. GitHub). See the Nature Research guidelines for submitting code & software for further information.

Data

Policy information about availability of data

All manuscripts must include a data availability statement. This statement should provide the following information, where applicable:

- Accession codes, unique identifiers, or web links for publicly available datasets
- A list of figures that have associated raw data
- A description of any restrictions on data availability

Raw data NGS counts are provided as Supplementary Data 7 and Supplementary Data 8 on files made using Excel 2016 (16.0.5032.1000)

Field-specific reporting

Please select the one below that is the best fit for your research. If you are not sure, read the appropriate sections before making your selection.

[x] Life sciences [ ] Behavioural & social sciences [ ] Ecological, evolutionary & environmental sciences

For a reference copy of the document with all sections, see nature.com/documents/nr-reporting-summary-flat.pdf

Life sciences study design

All studies must disclose on these points even when the disclosure is negative.

Sample size

Pooled CRISPR screening used 2 independent technical replicates with high coverage (500-1000 cells per sgRNA). This 500x -1000x coverage per sgRNA provides high sensitivity to detect small changes in fitness in an internally controlled setting. Due to the high volume of cells only 2 technical replicates were possible per screen, but different cell clones were tested and screens were also repeated using 2-3 different Cas9 editing time points. For mini-pool validation studies 6 independent technical replicates were performed per cell line per time point.

Love, M. I., Huber, W. & Anders, S. Moderated estimation of fold change and dispersion for RNA-seq data with DESeq2. Genome Biol. 15, 550 (2014).

For neuronal validation experiments to determine hit significance, 7 biological replicates were performed. Tau protein levels in each well were first normalized to total protein per well. Each replicate plate was normalized to its own control wells to account for inter-plate variability. The results of the normalizations for all 7 individual replicates were then pooled and the median of the normalized total tau was calculated per gRNA. Z-scores for each gRNA were then calculated using the normalized medians by calculating the mean and standard deviation of the whole set.

For rat neuronal tau aggregation assay to determine if hits affected tau aggregation levels, 4 biological replicates were performed. Images were analyzed for inclusion counts using Cell Profiler. 9 fields were taken per well and each field was then analyzed to determine inclusion (tau aggregate) count and nuclear counts. All fields per well were then pooled to determine the total number of inclusions and nucleus quantified per well. Background of stain was filtered out by comparing wells with no seed controls and determining how many inclusions are picked up by cell profiler in these wells. The total number of inclusions per well were then normalized to the number of inclusions in non-target control wells to determine fold change relative to non-target controls.

For mouse work sample size for transgenic animals was determined based on previous data, demonstrating that minimally an n=6 required to properly power for modulation of mTOR pathway activation.

Data exclusions

No data was excluded

Replication

For primary and validation pooled CRISPR screens 2 technical replicates were performed per screen per cell clone and per editing time point. For mini-pool validation studies 6 independent technical replicates were performed per cell line per time point. For neuronal validation experiments 7 independent biological replicates were performed. All data was replicated.
For rat neuronal tau aggregation assay 4 independent biological replicates were performed. All data was replicated.

Randomization

Sample randomization is not applicable for in vitro cell culture pooled CRISPR screening experiments where large batches of homogeneous cultures can be easily tested in parallel. We went to great lengths to minimize the potential effects of a particular reagent such as a gRNAs, Cas9 expression system, or genetic background.

Phenotypic, molecular (AlphaLISA) and Cas9 genome engineering assays were used in H1-hESCs (human embryonic stem cells) differentiated into neurons using two distinct gRNAs targeting each gene to be validated.

For mouse work all animals used in study were naive requiring no randomization.

Blinding

All computational analysis for the screen was conducted by bio-informaticians not involved the design of the study. For this reason they had no preconceptions about the data and were therefore blind to the outcome. This types of analysis applies to pooled CRISPR analysis, STRING analysis and Next Generation Sequencing analysis.

Reporting for specific materials, systems and methods

We require information from authors about some types of materials, experimental systems and methods used in many studies. Here, indicate whether each material, system or method listed is relevant to your study. If you are not sure if a list item applies to your research, read the appropriate section before selecting a response.

Materials & experimental systems

n/a	Involved in the study
☐	☒ Antibodies
☐	☒ Eukaryotic cell lines
☒	☐ Palaeontology and archaeology
☐	☒ Animals and other organisms
☒	☐ Human research participants
☒	☐ Clinical data
☒	☐ Dual use research of concern

Methods

n/a	Involved in the study
☒	☐ ChIP-seq
☐	☒ Flow cytometry
☒	☐ MRI-based neuroimaging

Antibodies

Antibodies used	The following primary antibodies were used to analyze in vitro samples: SP70 (ThermoFisher Scientific #MA5-16404, 1:1000), Cas9 (Diagenode #C15200229, 1:400), M2 FLAG (Sigma # F1804, 1:500), β-actin (Sigma #A5316, 1:1000), Tubb1 (Bio Legend #801201, 1:1000), Map2 (LS-Bio #LS-C61805, 1:1000), Oct4 (Stem Cell #60093, 1:500), Anti-Tau (3-repeat isoform RD3; Millipore #05-803, 1:100), Anti-Tau (4-repeat isoform RD4; Millipore #05-804, 1:100), Anti-Tau Antibody, clone T49 (Not human; Millipore # MABN827, 1:1000) . The following primary antibodies were used to analyze total protein or phosphorylation levels in in vivo studies: Total S6 (Cell Signaling #2317, 1:500), pS6 Ser240/244 (Cell Signaling #5364, 1:2000).
Validation	Tau protein antibodies T49, SP70, HT7 and BT2 antibodies were validated in the manuscript (Figures 1, Supplementary Figure 1, Figure 3 and Supplementary Figure 4) by using tau protein knockouts. Cas9 and FLAG antibodies were validated by introduction of Cas9 transgenes into cell lines and comparing to parental cell lines without Cas9 transgene. Tubb1, Map2 and Oct4 were validated by expression, or no expression, in differentiated neuronal cells (Supplementary Figure 3). All other antibodies used in the study were validated by manufacturer.

Eukaryotic cell lines

Policy information about cell lines

Cell line source(s)	SH-SY5Y (ATCC CRL-2266), HEK 293T (AF-87-QC67), H1 hESC (NIHhESC-10-0043; WAe001-A), Rat cortical neurons
Authentication	H1-hESCs were authenticated by SNP fingerprinting, Karyotype analysis and staining for pluripotent stem cell markers.
Mycoplasma contamination	Cells were not tested for mycoplasma contamination
Commonly misidentified lines (See ICLAC register)	None

Animals and other organisms

Policy information about studies involving animals; ARRIVE guidelines recommended for reporting animal research

Laboratory animals	Tsc1 fl/fl CamKIIα-Cre; mixed gender
Wild animals	Study did not involve wild animals.
Field-collected samples	All animals were housed with a regulated temperature and light cycle (22 °C, 12 h light/12 h darkcycle) with unrestricted access to food and water.
Ethics oversight	All animal experiments were performed in accordance with institutional guidelines for the care and use of laboratory animals as approved by the Institutional Animal Care and Use Committee (IACUC) of the Novartis Institutes for BioMedical Research, Inc. (Cambridge, MA).

Note that full information on the approval of the study protocol must also be provided in the manuscript.

Flow Cytometry

Plots

Confirm that:

- [x] The axis labels state the marker and fluorochrome used (e.g. CD4-FITC).
- [x] The axis scales are clearly visible. Include numbers along axes only for bottom left plot of group (a 'group' is an analysis of identical markers).
- [x] All plots are contour plots with outliers or pseudocolor plots.
- [x] A numerical value for number of cells or percentage (with statistics) is provided.

Methodology

Sample preparation	Cells were trypsinized, fixed and stained for tau using SP70. Cells were fixed in 4% paraformaldehyde for 20 minutes, then washed with PBS, permeabilized in 0.1% Triton X-100 for 10min, blocked using Odyssey block buffer (LI-COR, #927-70001) for 30minutes, and incubated with SP70 primary antibody at 1:100 in 1:1 PBS/Odyssey block buffer for 1hour at room temperature. Then cells were washed with PBS 2-3 times and incubated with Alexa647-tagged secondary antibody (ThermoFisher Scientific #A32733) in the dark for 30min at room temperature. Finally, cells were washed 2-3 times with PBS and strained through a 40µm mesh filter before re-suspending at 30 million cells/mL. Prior to FACS, 1.5 mM EDTA (ThermoFisher Scientific #R1021) was spiked into wash buffers and cells were always kept in ice-cold PBS to avoid cell clumping. As a starting point 2 billion cells were used for tau antibody staining. Because many cells are lost during staining procedure, this yielded 600-800M cells for FACS sorting. In order to minimize cell loss from cells sticking to tubes, all tubes were pre-coated with FBS (ThermoFisher Scientific #10438026).
Instrument	BD FACS ARIA III
Software	FlowJo 10.6.0
Cell population abundance	For primary screens 50 million cells were collected per population, for validation screens 2 million cells were collected per population.
Gating strategy	Cells were gated based on 647 fluorescence after immunocytochemistry. The 25% low 647 cells, and the 25% high 647 cells were sorted as distinct cell populations.

- [ ] Tick this box to confirm that a figure exemplifying the gating strategy is provided in the Supplementary Information.
